# Cigarette smoke causes a bioenergetic crisis in RPE cells involving the downregulation of HIF-1α under normoxia

**DOI:** 10.1038/s41420-023-01695-5

**Published:** 2023-10-25

**Authors:** Yoshiyuki Henning, Katrin Willbrand, Safa Larafa, Gesa Weißenberg, Veronika Matschke, Carsten Theiss, Gina-Eva Görtz, Johann Matschke

**Affiliations:** 1 Institute of Physiology, University Hospital Essen, University of Duisburg-Essen, Essen, Germany; 2 Institute of Cell Biology (Cancer Research), University Hospital Essen, University of Duisburg-Essen, Essen, Germany; 3 Department of Cytology, Institute of Anatomy, Ruhr University Bochum, Bochum, Germany; 4 Molecular Ophthalmology, Department of Ophthalmology, University Hospital Essen, University of Duisburg-Essen, Essen, Germany

**Keywords:** Cell death, Mechanisms of disease

## Abstract

Age-related macular degeneration (AMD) is the most common blinding disease in the elderly population. However, there are still many uncertainties regarding the pathophysiology at the molecular level. Currently, impaired energy metabolism in retinal pigment epithelium (RPE) cells is discussed as one major hallmark of early AMD pathophysiology. Hypoxia-inducible factors (HIFs) are important modulators of mitochondrial function. Moreover, smoking is the most important modifiable risk factor for AMD and is known to impair mitochondrial integrity. Therefore, our aim was to establish a cell-based assay that enables us to investigate how smoking affects mitochondrial function in conjunction with HIF signaling in RPE cells. For this purpose, we treated a human RPE cell line with cigarette smoke extract (CSE) under normoxia (21% O_2_), hypoxia (1% O_2_), or by co-treatment with Roxadustat, a clinically approved HIF stabilizer. CSE treatment impaired mitochondrial integrity, involving increased mitochondrial reactive oxygen species, disruption of mitochondrial membrane potential, and altered mitochondrial morphology. Treatment effects on cell metabolism were analyzed using a Seahorse Bioanalyzer. Mitochondrial respiration and ATP production were impaired in CSE-treated cells under normoxia. Surprisingly, CSE-treated RPE cells also exhibited decreased glycolytic rate under normoxia, causing a bioenergetic crisis, because two major metabolic pathways that provide ATP were impaired by CSE. Downregulation of glycolytic rate was HIF-dependent because HIF-1α, the α-subunit of HIF-1, was downregulated by CSE on the protein level, especially under normoxia. Moreover, hypoxia incubation and treatment with Roxadustat restored glycolytic flux. Taken together, our in vitro model provides interesting insights into HIF-dependent regulation of glycolysis under normoxic conditions, which will enable us to investigate signaling pathways involved in RPE metabolism in health and disease.

## Introduction

Retinal pigment epithelium (RPE) cells, a monolayer of pigmented cells located adjacent to the photoreceptors, serve as a gatekeeper, which regulates the transport of nutrients, oxygen, and ions from the choriocapillaris to the photoreceptors as well as the disposal of metabolic end-products [1]. In addition, RPE cells protect photoreceptors from light-induced damage, support the renewal of photoreceptor outer segments by phagocytosis, and execute the visual cycle, to mention a few functions [1]. Furthermore, there is a fine-tuned metabolic interaction of RPE cells and photoreceptors. RPE cells, which mainly produce ATP by mitochondrial respiration, supply glucose to the photoreceptors. Photoreceptors, in turn, generate energy mainly by aerobic glycolysis and supply the RPE cells with the resulting lactate to fuel mitochondrial respiration [2, 3]. Consequently, dysregulation of the photoreceptor–RPE interaction results in visual disorders, such as age-related macular degeneration (AMD). AMD is the most common blinding disease in industrial countries diagnosed in people of 60 years and older. Globally, it represents the third leading cause of irreversible vision loss worldwide with increasing prevalence [4–6]. In the later stages of AMD, about 90% of the patients develop dry AMD or geographic atrophy, which is associated with dysfunction and gradual degeneration of RPE cells, choriocapillaris, and photoreceptors. There is still no convincing treatment for dry AMD due to a lack of mechanistic understanding driving the disease [7]. Thus, to develop novel treatment options, a deeper understanding of early AMD pathophysiology and its risk factors is necessary.

Major hallmarks often observed in early AMD are thickening of the Bruch’s membrane, reduced choroidal blood flow, and the appearance of drusen, deposits of metabolic products accumulating between the RPE and Bruch’s membrane [6, 8–10]. These structural changes impair the oxygen supply of the RPE and photoreceptors, resulting in hypoxia, a state where oxygen demand exceeds the supply. Hypoxia results in the stabilization of hypoxia-inducible factors (HIFs), which regulate the adaptation to hypoxic conditions by, e.g. initiating a metabolic switch from oxidative phosphorylation (OXPHOS) to glycolysis or expression of proangiogenic growth factors. HIFs are dimeric transcription factors with an oxygen-labile α-subunit and a constitutively expressed β-subunit located in the nucleus [11, 12]. When oxygen is available at physiological levels, α-subunits are constantly degraded after hydroxylation by oxygen-dependent prolyl hydroxylases 1–3 (PHD1, PHD2, and PHD3) [13]. To date three HIF isoforms are known from which HIF-1 and HIF-2 are considered most relevant for the hypoxia response [14]. HIFs are protective in the short-term, but chronic HIF stabilization in the RPE can exert detrimental effects [15–18], from which the metabolic shift is most relevant for the present study. Interestingly, there is increasing evidence from different models, including human AMD donor cells, that dysregulation of RPE energy metabolism contributes to AMD pathophysiology [19–25]. Accordingly, improvement of mitochondrial function has been proposed as an early intervention strategy against dry AMD [26, 27].

The many risk factors contributing to the development of AMD are divided into non-modifiable and modifiable risk factors. While the age of a person is the most important non-modifiable risk factor, smoking is the most important modifiable risk factor [9]. Cigarette smoke or cigarette smoke extract (CSE), which is used in cell culture experiments, promotes several pathophysiological processes associated with AMD in RPE and surrounding tissues, including oxidative stress, inflammation, cell senescence, and neovascularization resulting in a significantly increased risk to develop AMD [4, 6, 9, 28]. Furthermore, CSE has adverse effects on mitochondrial integrity in RPE cells [29–31] as well as lung, airway, and bronchial epithelial cells [32–36]. Moreover, in cultured fibroblasts, CSE enhanced HIF-1α stabilization and HIF signaling [37]. Therefore, we hypothesized that CSE-induced impairment of mitochondrial function is regulated in a HIF-dependent manner and that targeting HIF might represent an interventional strategy against mitochondrial dysfunction. For this purpose, we developed a cell-based assay that enabled us to conduct a detailed characterization of energy metabolism and mitochondrial integrity of CSE-treated RPE cells and analyzed the involvement of HIF signaling in the metabolic phenotype of these cells.

## Results

### CSE-treated cells displayed reduced mitochondrial respiration and glycolytic capacity

**Fig. 1:**
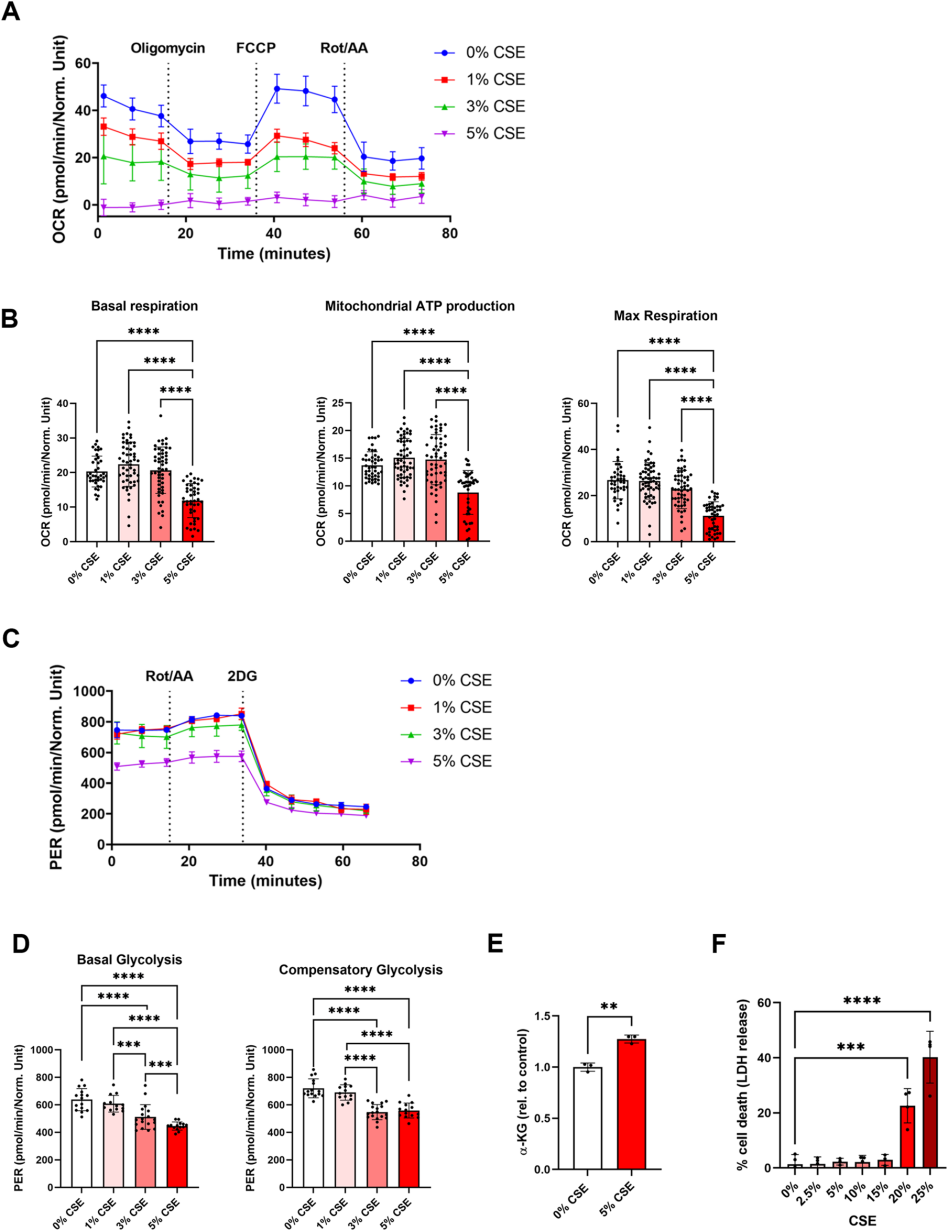
Mitochondrial respiration and glycolytic flux in CSE-treated ARPE-19 cells. **A** Mitochondrial oxygen consumption rate (OCR) representing oxidative phosphorylation in ARPE-19 cells treated with different CSE concentrations was determined by using a Mitochondrial Stress Test and a Seahorse XFe96 Analyzer. **B** Basal respiration, mitochondrial ATP production, and maximum respiration of CSE-treated ARPE-19 cells based on a Mitochondrial Stress Test. Data was obtained from *n* = 4–8 wells for each independent sample (*N* = 6–8 per treatment group). **C** Proton efflux rate (PER) representing glycolytic rate in ARPE-19 cells treated with different CSE concentrations determined with a Glycolysis Rate Assay using a Seahorse XFe96 Analyzer. **D** Basal and compensatory glycolysis of CSE-treated ARPE-19 cells based on a Glycolysis Stress Test. Data was obtained from *n* = 4–8 wells for each independent sample (*N* = 3 per treatment group). **E** Measurement of α-ketoglutarate (α-KG) in CSE-treated cells expressed relative to untreated control cells (*N* = 3). **F** LDH release as a measure of cell death from cells treated with different CSE concentrations. Seahorse and LDH datasets were statistically analyzed with one-way ANOVA followed by Tukey’s multiple comparisons test and α-ketoglutarate measurement was analyzed with unpaired *t*-test. **p < 0.01, ***p < 0.001, and ****p < 0.0001.

In order to investigate how CSE affects mitochondrial function, we analyzed oxidative phosphorylation (OXPHOS) by using a Seahorse XFe96 Analyzer (Fig. 1A). In particular, we determined basal respiration, mitochondrial ATP production, and maximal respiration (Fig. 1B) under normoxic conditions (21% O_2_) after cells were treated with CSE for 16 h. All parameters were decreased by CSE-treatment in a concentration-dependent manner. In the next step, we analyzed glycolysis, which usually compensates for reduced mitochondrial ATP production by OXPHOS (Fig. 1C). However, both, basal glycolysis as well as compensatory glycolysis were decreased by CSE in a concentration-dependent manner (Fig. 1D). Next, we measured α-ketoglutarate concentrations, an intermediate of the tricarboxylic acid (TCA) cycle. We found that α-ketoglutarate concentrations in ARPE-19 cells treated with 5% CSE were significantly higher compared to controls (Fig. 1E). To exclude the possibility that treatment with 5% CSE impairs cell viability, leading to the observed effects, we measured LDH release, which was not affected by CSE treatment up to a CSE concentration of 15% (Fig. 1F). Taken together, overall ATP production by mitochondrial respiration and glycolysis was impaired by CSE treatment.

### CSE treatment reduced HIF-1α protein levels via differential mechanisms depending on oxygen levels

**Fig. 2:**
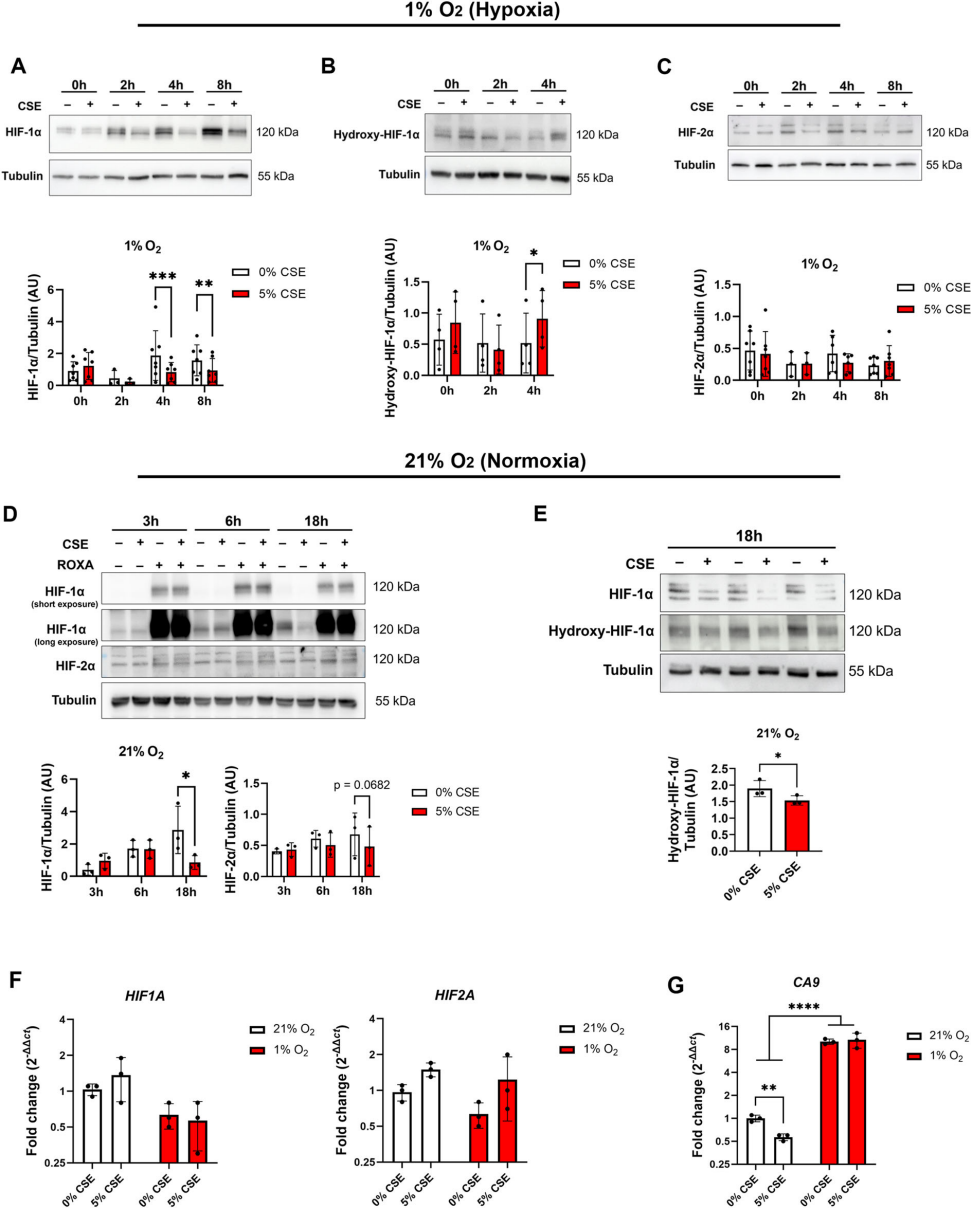
Quantification of HIF protein and mRNA expression levels under normoxic and hypoxic conditions. Representative images and statistical analyses of **A** HIF-1α, **B** hydroxy-HIF-1α, and **C** HIF-2α protein levels normalized to α-Tubulin in ARPE-19 cells treated with 5% CSE at 1% O_2_ compared to controls assessed at different timepoints (*N* = 3–7). **D** Representative images and statistical analyses of HIF-1α and HIF-2α protein levels normalized to α-Tubulin in ARPE-19 cells treated with 5% CSE compared to controls assessed at different timepoints at 21% O_2_ with or without 30 µM Roxadustat (ROXA) as positive control. To depict HIF-1α bands in cells treated with ROXA, a short exposure time was chosen (<5 s). To depict HIF-1α bands in cells treated at 21% O_2_ without ROXA, a long exposure time (>2 min) was chosen because HIF-1α is rapidly degraded under normoxia. **E** Representative images and statistical analyses of hydroxy-HIF-1α protein levels normalized to α-Tubulin in ARPE-19 cells from three independent groups treated with 5% CSE for 18 h at 21% O_2_. HIF-1α is shown above to show downregulation of HIF-1α by CSE (*N* = 3). **F** Gene expression of *HIF1A* (left panel), *HIF2A* (right panel), and **G** *Carbonic Anhydrase 9* (*CA9*) in CSE-treated ARPE-19 cells under normoxia and hypoxia determined by qRT-PCR expressed as 2^−∆∆ct^ (*N* = 3). Western blot data were statistically analyzed with two-way ANOVA followed by Šídák’s multiple comparisons test and qRT-PCR data were analyzed by two-way ANOVA followed by Tukey’s multiple comparisons test. AU arbitrary units. *p < 0.05, **p < 0.01, ***p < 0.001, and ****p < 0.0001.

HIFs are one of the main regulators of glycolysis-driven ATP production. Therefore, we measured HIF protein levels in ARPE-19 cells treated with 5% CSE in a time-course experiment in hypoxia and normoxia. We observed significantly decreased HIF-1α protein levels after 4 and 8 h of CSE treatment under hypoxia (1% O_2_) (Fig. 2A). Furthermore, we measured hydroxylated HIF-1α levels under the same conditions as a measure of hydroxylation by PHDs and subsequent degradation, which was significantly upregulated after 4 h (Fig. 2B). In contrast, HIF-2α protein levels remained unchanged under hypoxic conditions (Fig. 2C). Under normoxic conditions (21% O_2_), HIF-1α levels were significantly downregulated by 5% CSE after 18 h (Fig. 2D). HIF-2α showed a trend towards downregulation by CSE but the effect was not as pronounced as for HIF-1α (Fig. 2D). In addition, PHD inhibition by Roxadustat served as a positive control for HIF bands under normoxia (Fig. 2D). In contrast to hypoxic conditions, hydroxylated HIF-1α levels were significantly lower in cell treated with 5% CSE (Fig. 2E). *HIF1A* and *HIF2A* mRNA expression was not affected by CSE (Fig. 2F). To test downstream effects of HIF-1α destabilization, we measured gene expression of the *Carbonic Anhydrase 9* (*CA9*), a specific target gene of HIF-1. *CA9* expression was downregulated by CSE treatment under normoxia but not hypoxia (Fig. 2G).

### CSE treatment impaired mitochondrial integrity

**Fig. 3:**
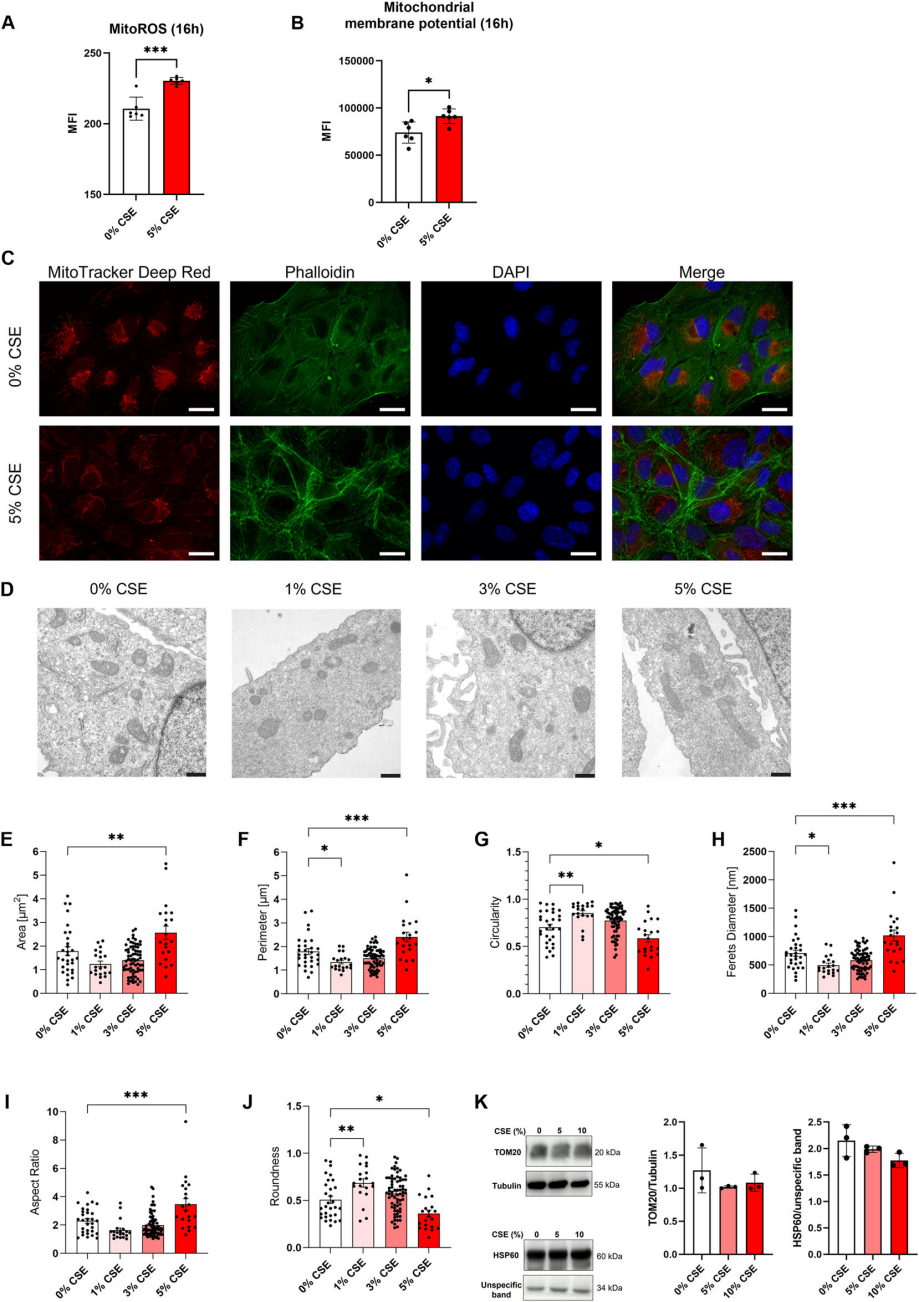
Analysis of mitochondrial integrity of CSE-treated ARPE-19 cells. **A** Mitochondrial ROS (MitoROS) and **B** mitochondrial membrane potential in ARPE-19 cells treated with 5% CSE for 16 h compared to untreated controls determined by flow cytometry and expressed as mean fluorescence intensity (MFI; *N* = 6). **C** Staining of ARPE-19 cells treated with 5% CSE for 8 h compared to untreated controls with MitoTracker Deep Red, a polarization-dependent dye to visualize mitochondrial membrane potential. Cells were counterstained with Phalloidin and DAPI. Scale bar 20 µm. **D** TEM micrographs of ARPE-19 cells treated with different CSE concentrations. **E**, **F** Mitochondrial area and perimeter of mitochondria from cells treated with different CSE concentrations as a measure of mitochondrial size (20–70 mitochondria of one independent sample per treatment group). **G**–**J** Circularity, ferrets diameter, aspect ratio, and roundness of mitochondria from cells treated with different CSE concentrations as a measure of mitochondrial shape (20–70 mitochondria of one independent sample per treatment group). **K** Western blot quantification of TOM20 and HSP60 protein levels as a measure of mitochondrial mass (*N* = 3). Flow cytometry data were statistically analyzed with two-way ANOVA followed by Šídák’s multiple comparisons test. TEM and Western Blot data were analyzed by one-way ANOVA followed by Tukey’s or Dunnett’s multiple comparisons test. *p < 0.05, **p < 0.01, ***p < 0.001, and ****p < 0.0001.

We treated ARPE-19 cells with CSE and measured mitochondrial reactive oxygen species (ROS) by flow cytometry. Mitochondrial ROS was significantly increased in CSE-treated cells after treatment with 5% CSE for 16 h under normoxia (Fig. 3A). Furthermore, we measured mitochondrial membrane potential in TMRE-stained cells by flow cytometry as a measure of mitochondrial membrane integrity. Compared to controls, mitochondrial membrane potential was upregulated (hyperpolarized) after treatment with 5% CSE for 16 h (Fig. 3B). In addition, we stained the cells with MitoTracker Deep Red, a polarization-dependent fluorescence staining of mitochondria [38, 39]. In cells treated with 5% CSE for 8 h, we found fainter and less pronounced staining of mitochondria, which confirmed mitochondrial depolarization detected by flow cytometry (Fig. 3C). For detailed examination of mitochondrial morphology in CSE-treated ARPE-19 cells, we conducted TEM analyses (Fig. 3D). Compared to controls, cells treated with 5% CSE displayed larger (Fig. 3E, F) and elongated (Fig. 3G–J) mitochondria compared to the respective non-treated controls. Mitochondrial mass, assessed by Western blot analysis of TOM20 and HSP60, was not significantly changed by CSE treatment (Fig. 3K).

### Hypoxia incubation and Roxadustat treatment restored glycolytic capacity

**Fig. 4:**
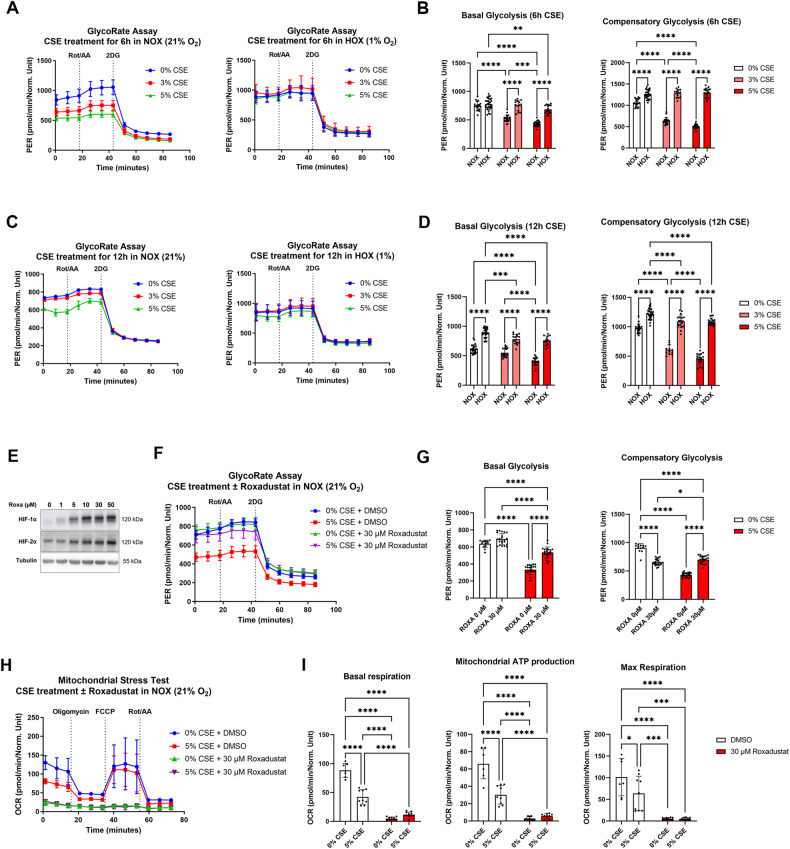
Restoration of glycolytic flux in CSE-treated ARPE-19 cells by hypoxia or Roxadustat treatments. **A** Proton efflux rate (PER) representing glycolysis rate in ARPE-19 cells treated with different CSE concentrations determined with a Glycolysis Rate Assay using a Seahorse XFe96 Analyzer. Cells were treated for 6 h with 5% CSE under 21% and 1% O_2_ with a 2 h preincubation under 21% or 1% O_2_ prior to CSE treatment. **B** Basal and compensatory glycolysis of CSE-treated ARPE-19 cells based on a Glycolysis Stress Test. Data was obtained from *n* = 4–13 wells for each independent sample (*N* = 3 per treatment group). **C** PER of ARPE-19 cells treated with different CSE concentrations. Cells were treated for 12 h with 5% CSE under 21% and 1% O_2_. **D** Basal and compensatory glycolysis of CSE-treated ARPE-19 cells. Data was obtained from *n* = 4–11 wells for each independent sample (*N* = 3 per treatment group). **E** Stabilization of HIF-1α and HIF-2α in ARPE-19 cells treated with different Roxadustat concentrations for 6 h (*N* = 3). **F** PER of ARPE-19 cells treated with 5% CSE and/or 30 µM Roxadustat. **G** Basal and compensatory glycolysis of ARPE-19 cells treated with 5% CSE with or without 30 µM Roxadustat (ROXA). Data was obtained from *n* = 2–8 wells for each independent sample (*N* = 3 per treatment group). **H** OCR of ARPE-19 cells treated with 5% CSE with or without 30 µM Roxadustat. **I** Basal respiration, mitochondrial ATP production, and maximum respiration of CSE/Roxadustat-treated ARPE-19 cells. Data was obtained from *n* = 7–11 wells for each independent sample (*N* = 3 per treatment group). Datasets were analyzed with two-way ANOVA followed by Tukey’s multiple comparisons test. *p < 0.05, **p < 0.01, ***p < 0.001, and ****p < 0.0001.

To determine whether metabolic reprogramming induced by CSE treatment and resulting HIF destabilization are related, we tested whether the glycolytic rate is restored after stabilization of HIF protein by hypoxia or Roxadustat. In the first approach, we treated ARPE-19 cells with 3% and 5% CSE for 6 h under normoxia and hypoxia (1% O_2_). The hypoxia group was additionally pre-incubated at 1% O_2_ for 2 h prior to CSE treatment (Fig. 4A, B). In a second approach, we treated ARPE-19 cells with CSE for 12 h under normoxia and hypoxia without pre-incubation (Fig. 4C, D). Both approaches significantly downregulated basal and compensatory glycolysis of CSE-treated cells under normoxia as observed in previous experiments. However, hypoxic treatment for either 6 or 12 h fully restored basal and compensatory glycolysis of CSE-treated ARPE-19 cells to the levels observed under normoxic conditions in non-treated cells (6 h: Fig. 4B; 12 h: Fig. 4D). Next, ARPE-19 cells were treated with Roxadustat, a specific PHD inhibitor, which stabilizes HIF-1α and HIF-2α in a concentration-dependent manner (Fig. 4E). Co-treatment with CSE and Roxadustat for 16 h significantly upregulated basal and compensatory glycolysis compared to CSE treatment alone (Fig. 4F, G), clearly indicating that CSE treatment impairs glycolytic capacity by fostering PHD-induced HIF degradation. As expected, OXPHOS was almost completely shut down by HIF stabilization using Roxadustat (Fig. 4H, I).

## Discussion

In the present study, we aimed to establish a cell-based assay that enables us to identify potential CSE effects and signaling pathways in RPE cells associated with metabolic dysregulation. We found that CSE treatment of ARPE-19 cells under normoxia resulted in downregulated basal respiration, mitochondrial ATP production, and maximum respiration, indicating impaired mitochondrial function. Usually, mitochondrial dysfunction results in increased glycolytic flux to prevent depletion of ATP [40–42]. Surprisingly, in CSE-treated ARPE-19 cells, we even observed a decreased glycolytic flux. Under physiological conditions, the HIF pathway tightly balances energy metabolism according to oxygen availability and other metabolic demands and as such it is one important regulator of the metabolic switch between mitochondrial respiration and glycolysis [43, 44]. However, despite downregulated OXPHOS, we found that HIF-1α protein levels were decreased in CSE-treated cells under normoxic and hypoxic conditions. Under normoxic conditions, CSE treatment led to the downregulation of *CA9*, a gene specifically targeted by HIF-1. This implies that the impact of CSE on HIF-1α is more evident under normoxic conditions, as the significant stabilization of HIF-1α in hypoxic conditions (due to O_2_-dependent inhibition of PHDs) could obscure the effect of CSE. This is further supported by the restoration of glycolytic flux under hypoxic conditions in a HIF-1-dependent manner. It might be surprising that the downregulation of HIF-1α under normoxic conditions had downstream effects on target gene expression and glycolysis in the present study because HIF-1 is usually considered a hypoxia-regulated transcription factor. However, it has been reported that HIF-1 regulates glycolysis also under normoxic conditions in several cell types [45–47]. The present study supports these findings as glycolysis in ARPE-19 cells was also shown to depend on HIF-1 signaling under normoxic conditions. Downregulation of HIF-1α is in good agreement with the lack of upregulation of glycolysis in our study but is in contrast to HIF-1α regulation reported in orbital fibroblasts, where HIF-1α protein levels and HIF signaling were both upregulated by CSE treatment [37]. In contrast, no significant CSE effects on HIF-2α were observed in our study. Downregulation of HIF-1α in CSE-treated ARPE-19 cells is likely to be caused at the posttranslational level since we found no changes in *HIF1A* mRNA expression. Interestingly, while downregulation of HIF-1α under hypoxia was accompanied by higher levels of hydroxylated HIF-1α, HIF-1α hydroxylation was even downregulated by CSE under normoxic conditions. We can infer from these data, that hydroxylation by PHDs is not involved in CSE-induced downregulation of HIF-1α under normoxia.

CSE treatment increased intracellular α-ketoglutarate concentrations, indicating inhibition of α-ketoglutarate dehydrogenase multienzyme complex (KGDHC), the rate-limiting enzyme complex of the TCA cycle [48]. This may be due to increased mitochondrial ROS levels observed in ARPE-19 cells, which were described to reduce KGDHC activity resulting in α-ketoglutarate accumulation [49]. Hydroxylation of HIF proline residues by PHDs requires α-ketoglutarate as a co-substrate [50]. Moreover, it has been shown that increased α-ketoglutarate levels promote PHD activity in various cell types, including RPE cells [51]. Thus, accumulation of α-ketoglutarate upon CSE treatment might lead to enhanced PHD activation resulting in increased HIF-degradation at least under hypoxia, where hydroxylated HIF-1α was increased by CSE. Interestingly, it has been shown that PHD2 has a higher affinity for α-ketoglutarate and HIF-1α compared to PHD3, which preferentially (but not exclusively) hydroxylates HIF-2α [52, 53]. This could explain why HIF-2α protein levels remained mostly unchanged upon CSE treatment, while HIF-1α protein levels significantly decreased in CSE-treated cells. On the other hand, decreased production of TCA cycle metabolites such as succinate and fumarate as a result of KGDHC inhibition by CSE treatment might reduce the transcriptional activity of HIFs also under normoxia [44, 54, 55].

Impairment of the electron transport chain (ETC) is one possible mechanism for increased mitochondrial ROS production via reverse electron transport to ETC complex 1 [56, 57], which could also explain why mitochondrial transmembrane potential was disturbed in CSE-treated ARPE-19 cells. It was reported that depolarization via mitochondrial uncoupling reduces mitochondrial ROS production and hyperpolarization promotes ROS generation and oxidative damage [58–61]. Unexpectedly, we observed enlarged, elongated mitochondria accompanied by no change in mitochondrial mass in ARPE-19 cells treated with 5% CSE compared to non-treated controls, which is an unusual phenotype when membrane potential is lost and ATP production ceases. Usually, such pathological changes of mitochondrial integrity would activate mitochondrial fission, which promotes mitophagy [62]. Thus far, it is unclear whether there is a causal relationship between mitochondrial transmembrane potential, ROS and mitochondrial morphology observed upon CSE-treatment in our study model. However, we can infer from our results that CSE treatment causes a marked disruption of mitochondrial integrity and, as a consequence, mitochondrial respiration. In accordance with these findings, Seahorse analyses revealed impaired OXPHOS in cells treated with 5% CSE without a compensatory switch to glycolysis, which would be expected in such an energy-deprived state [40–42]. We were able to restore glycolytic capacity by either incubating the cells under hypoxic conditions or treatment with Roxadustat, providing a direct link between CSE-dependent metabolic dysregulation and HIF-1α degradation under normoxia. Future research should also address whether CSE-induced downregulation of glycolytic flux is caused by disruption of the transcriptional activity of HIF-1 or by a recently reported mechanism by which HIF-1α is involved in the cytoplasmic formation of a metabolic complex involving several glycolytic enzymes [63].

**Fig. 5:**
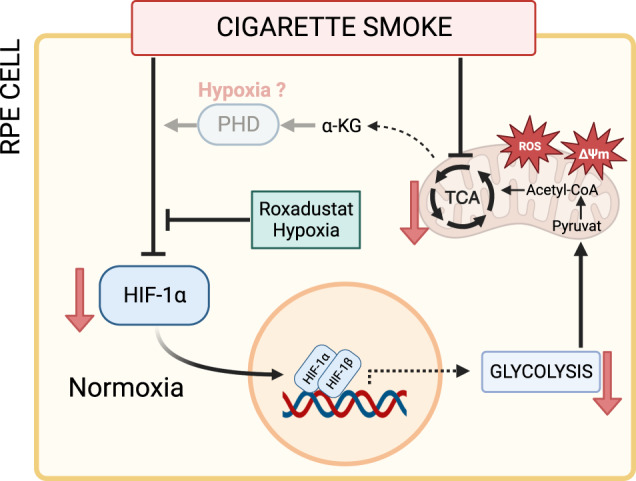
Conclusion. In the present study, treatment of ARPE-19 cells with CSE was shown to downregulate mitochondrial respiration, presumably by TCA cycle inhibition indicated by increased α-ketoglutarate (α-KG) levels in CSE-treated cells under normoxic conditions (21% O_2_). CSE treatment also affected mitochondrial morphology and increased mitochondrial ROS as well as mitochondrial membrane potential (hyperpolarization). Glycolysis could not fully compensate for impaired mitochondrial respiration, because CSE inhibited glycolysis indirectly by downregulating HIF-1α protein levels under normoxia, resulting in a bioenergetic crisis, because two major metabolic pathways to generate ATP are inhibited by CSE. At least glycolysis was restored by incubation of cells under hypoxia (1% O_2_) or with Roxadustat, which both induce HIF stabilization. These findings point out that HIF-1 is also responsible for the regulation of glycolysis under normoxic conditions in ARPE-19 cells. HIF-1α was also downregulated by CSE under hypoxic conditions, but downstream effects of CSE-induced HIF-1α downregulation were only observed under normoxic conditions. Moreover, while downregulation of HIF-1α under hypoxia was accompanied by increased HIF-1α hydroxylation by PHDs (possibly induced by higher α-KG levels), hydroxy-HIF-1α was even downregulated by CSE under normoxic conditions, suggesting that CSE acts differentially on HIF-1α protein depending on oxygen availability. Red arrows depict CSE-induced effects. The figure was created with BioRender.com.

In summary, we found dysregulated HIF signaling in CSE-treated cells under normoxic conditions, which also had functional implications for ARPE-19 cells as not only OXPHOS but also glycolysis was impaired. While impairment of OXPHOS was not HIF-dependent, the lack of a compensatory switch to glycolysis was caused by HIF destabilization under normoxia (Fig. 5). Most interestingly, there is still not much known about the role and regulation of HIFs under normoxic conditions beyond cancer cells and our findings provide important data on the regulation of glycolysis under the control of HIFs under normoxic conditions. In healthy RPE cells, mitochondrial respiration is the main source of ATP synthesis. Therefore, upregulating the glycolytic rate without restoring OXPHOS, as conducted in this study, would result in two major problems when translated into the in vivo situation. First, glycolysis alone is not sufficient to cover the metabolic demand of RPE cells, and second, RPE cells would metabolize far more glucose to fuel their own glycolysis, which would lead to a glucose shortage in glucose-dependent photoreceptor cells. Investigation of these systemic associations will be the subject of future studies.

## Material and methods

### Cell culture

Human RPE cells (ARPE-19, CRL-2302, American Type Culture Collection, Manassas, VA; distributed by LGC Standards GmbH, Wesel, Germany) were routinely cultivated in Dulbecco’s modified Eagle’s medium (DMEM)/F-12 (11330057, Thermo Fisher Scientific), supplemented with 10 % FBS and penicillin–streptomycin in a humidified incubator at 37 °C with 5% CO_2_. All experiments were conducted with DMEM/F12 supplemented with 1% FBS.

Cells were authenticated by STR profiling and routinely tested for mycoplasma contamination. All experiments were conducted within five passages.

### HIF stabilization

For HIF stabilization, cells were either treated under hypoxia or with Roxadustat, a clinically approved PHD inhibitor [64], under normoxia (21% O_2_). Hypoxia was achieved by incubating the cells in a hypoxic chamber at 1% O_2_ for the indicated time. For Roxadustat treatment, a stock solution of 10 mM in DMSO was diluted to a final concentration of 30 µM in a cell culture medium.

### Preparation of cigarette smoke extract

CSE was prepared by bubbling the smoke from four filter cigarettes (Marlboro; Philip Morris Products, Neuchatel, Switzerland; nicotine 0.9 mg, tar 12 mg) through 30 ml cell culture medium without additives (DMEM/F-12 with GlutaMax, Gibco) with a vacuum pump. Each cigarette was smoked in 10 puffs with 30 s breaks. 75% of each cigarette was smoked. This corresponds to a smoke length of 4.7 cm and a tobacco quantity of 530 mg with a theoretical nicotine content of 53.2 mg. The exposure quantity calculation was based on the results of Bernhard et al. [65].

### Western blot

Cells were seeded in six-well plates at a density of 200,000 cells/well and grown to confluence. For protein isolation, cells were washed with cold PBS, collected in lysis buffer using a cell scraper, incubated on ice for 20 min, and centrifuged for 5 min at 500 rpm. The supernatant was stored at −80 °C until use. 30 µg of total protein were incubated in Laemmli sample buffer for 5 min at 95 °C and subjected to SDS–PAGE. Separated proteins were transferred to a PVDF membrane with a Trans-Blot Turbo Transfer System (Bio-Rad Laboratories, Feldkirchen, Germany). Membranes were blocked with 5% skim milk in TBS-T for 1 h at room temperature. Primary antibodies against HIF-1α (610958, BD Biosciences, Franklin Lakes, NJ), hydroxy-HIF-1α (3434, Cell Signaling Technology, Danvers, MA), HIF-2α (NB100-122, Novus Biologicals, Littleton, CO), TOM20 (11802-1-AP, Proteintech Group, Rosemont, IL), HSP60 (15282-1-AP, Proteintech Group), and Tubulin (sc-8035, Santa Cruz Biotechnology, Dallas, TX) were diluted in blocking buffer and incubated overnight at 4 °C. To detect proteins, goat anti-mouse (A2304, Sigma Aldrich, St. Louis, MO, USA) and goat anti-rabbit (A0545, Sigma Aldrich) secondary antibodies were diluted in a blocking buffer and incubated for 1 h at room temperature. Signals were developed with SuperSignal West Femto Maximum Sensitivity Substrate (34096, Thermo Fisher Scientific), detected with a Fusion FX System (Vilber, Eberhardzell, Germany), and quantified with ImageJ. Full-length uncropped original western blots are provided in Supplemental Material (Supplementary File 1).

### Quantitative real-time PCR

Total RNA was isolated using the NucleoSpin RNA kit (740955.250, Macherey-Nagel, Düren Germany) according to the manufacturer’s instructions. Complementary DNA (cDNA) was synthesized from 500 ng total RNA using M-MLV reverse transcriptase (M1705, Promega, Walldorf, Germany) and oligo dT primer. Quantitative Real-Time PCR (qRT-PCR) was performed with a Biozym Blue S’Green master mix (331416XL, Biozym Scientific, Hessisch Oldendorf, Germany) on a BioRad CFX Opus 96 System. Relative expression levels were calculated with the ΔΔct method using *hypoxanthine-guanine phosphoribosyltransferase* (*HPRT*) as a reference gene. The following primer pairs were used: *HIF1A* 5′-GGATGCTGGTGATTTGGATA-3′ (forward) and 5′-TCATGGTCACATGGATGAGTA-3′ (reverse); *HIF2A* 5′-CGGAGGTGTTCTATGAGCTGG-3′ (forward) and 5′-AGCTTGTGTGTTCGCAGGAA-3′ (reverse); *CA9* 5′-CACGTGGTTCACCTCAGCAC-3′ (forward) and 5′-CAGCGATTTCTTCCAAGCG-3′ (reverse); *HPRT* 5′-CCTGGCGTCGTGATTAGTGA-3′ (forward) and 5′-CGAGCAAGACGTTCAGTCCT-3′ (reverse).

### Flow cytometry analysis of reactive oxygen species and mitochondrial membrane potential

Cells were plated in six-well plates at a density of 200,000 cells/well. Confluent cells were treated with CSE for indicated time periods. The supernatants were collected in flow cytometry tubes before detaching the cells with accutase (PAN Biotech, Germany). Accutase reaction was stopped by the addition of cell culture media. Detached cells were transferred to flow cytometry tubes and centrifuged at 1500 rpm at room temperature. The supernatant was discarded and cell pellets were separately stained with the following staining solutions for different purposes as previously described [66–68]. Mitochondrial ROS production was assessed by staining the cells with 5 μM of MitoSOX (Molecular Probes/Invitrogen, Carlsbad, CA, USA) for 30 min at 37 °C. To analyze mitochondrial membrane potential, cell pellets were stained using 25 nM of the potential-specific dye tetramethyl rhodamine ethyl ester (TMRE; Molecular Probes/Invitrogen, Carlsbad, CA, USA) for 30 min at 37 °C as previously described [69]. TMRE staining allows for measuring dissipation of the mitochondrial membrane potential (ΔΨm) as depolarized or inactive mitochondria have decreased membrane potential and fail to bind TMRE. Readouts were accessed by flow cytometry (CytoFLEX S, Beckman Coulter).

### MitoTracker deep red staining

To visualize mitochondrial membrane potential, cells were treated with 5% CSE for 8 h and afterward incubated with fresh cell culture medium containing MitoTracker Deep Red FM (1:5000; Molecular Probes/Invitrogen, Carlsbad, CA, USA), a polarization-dependent fluorescence staining of mitochondria [38, 39], for 15 min in the dark. Cells were washed and fixed with 3% paraformaldehyde containing 0.2% Triton X-100 in PBS. For counterstaining, cells were incubated with Phalloidin-TRITC (1:200) and embedded with Mowiol containing DAPI (1:1000). Fluorescence imaging was conducted with a Zeiss Axio Observer Z1 fluorescence microscope with ApoTome and ZEN imaging software (Carl Zeiss, Goettingen, Germany).

### Extracellular flux assay—mitochondrial stress test

Cells were plated at a density of 7500 cells/well in XF96 micro-plates (Agilent Technologies, Santa Clara, CA, USA) in DMEM/F12 supplemented with 10% FBS 48 h prior to the assay and cultivated in a humified incubator at 37 °C with 5% CO_2_. All treatments were conducted in DMEM/F12 with 1% FBS for the indicated time. 1 h prior to the assay time point, the medium was exchanged to the Seahorse XF Base Medium (Agilent Technologies) with 1 mM sodium pyruvate, 2 mM l-glutamine and 10 mM d-(+)-glucose (all Sigma-Aldrich), pH 7.4 and incubated at 37 °C without CO_2_. During assays, oxygen consumption rate (OCR) and extracellular acidification rate (ECAR) were measured in parallel using a Seahorse XFe96 Analyzer (Agilent Technologies). Seahorse XF Cell Mito Stress Test kit (Agilent Technologies) containing injections of oligomycin (1 µM), carbonyl cyanide-4-(trifluoromethoxy) phenylhydrazone (FCCP, 0.25 µM), followed by combined injection of rotenone and antimycin A (0.5 µM) was performed according to manufacturer’s protocol. For individual well normalization of cell number, DNA content fluorescence was measured after cells were stained with 10 mg/mL Hoechst 33342 (Sigma-Aldrich) solution after each assay. Data were analyzed using Wave 2.6 software (Agilent Technologies). Mean values of *n* = 13–52 wells per experimental group from a total *N* = 3–7 independent experiments were used for the analysis as previously described [70, 71]. All metabolic parameters were normalized to Hoechst intensity (relative fluorescence units, RFU) in each well.

### Extracellular flux assay—glycolysis rate assay

Cells were plated at a density of 7500 cells/well in XF96 micro-plates (Agilent Technologies) in DMEM/F12 supplemented with 10% FBS 48 h prior to the assay and cultivated in a humified incubator at 37 °C with 5% CO_2_. All treatments were conducted in DMEM/F12 with 1% FBS for the indicated time. For Glycolytic Rate Assay, the medium was exchanged to XF DMEM medium (Agilent Technologies) with 2 mM glutamine, 10 mM glucose, 1 mM pyruvate, and 5 mM HEPES and incubated at 37 °C without CO_2_ 1 h prior to the assay. During the assay, the extracellular acidification rate (ECAR) was measured using a Seahorse XFe96 Analyzer. Glycolytic Rate Assay Kit containing 0.5 μM Rotenone, 0.5 μM Antimycin A, and 50 mM 2-Desoxyglucose was performed according to the manufacturer’s protocol. Proton efflux rate (PER) was calculated by Wave 2.6 (Agilent Technologies) software after the assay. Normalization of the cell number and data evaluation using Wave 2.6 software was performed as described above for the mitochondrial stress test.

### LDH assay

To assess cell viability, LDH release was measured using a CyQUANT™ LDH Cytotoxicity Assay (C20301, Thermo Fisher Scientific). Briefly, cells were seeded at a density of 10,000 cells/well in a 96-well plate. Confluent cells were treated with different CSE concentrations for 24 h. Subsequently, 50 µL of cell culture medium was transferred to a 96-well plate and subjected to LDH measurement according to the manufacturer’s instructions. Absorbance was measured at 490 nm with a plate reader.

### Measurement of α-ketoglutarate

Intracellular α-ketoglutarate was measured using a colorimetric α-ketoglutarate assay (MET-5131, Cell Biolabs Inc., San Diego, CA) according to the manufacturer’s instructions. In brief, ARPE-19 cells were treated with 5% CSE for 24 h and subsequently homogenized in assay buffer before centrifugation at 12,000×*g* for 10 min to collect the supernatant. To avoid oxidation of the samples, superoxide dismutase (S9697, Sigma Aldrich) at a final concentration of 40 U/mL was added to the samples. Samples were incubated for 2 h with the reaction reagent and absorbance was measured at 540 nm.

### Transmission electron microscopy

For transmission electron microscopy (TEM), ARPE-19 cells were seeded in T25 flasks and grown to confluence. Confluent cells were treated with 0%, 1%, 3%, and 5% CSE for 16 h. Subsequently, after treatment, cells were fixed with 2% glutaraldehyde (#G5882, Merck, Darmstadt, Germany) in 0.1 M cacodylate buffer for 1.5 h. After cells were washed with phosphate buffer (PB), samples were stained with 1% osmium tetroxide in H_2_O for 1 h. Thereafter, cells were detached from the flask’s bottom with cell scrapers and centrifuged at 1041×*g* for 10 min. Afterward, cell pellets were embedded in Agar–Agar (4% in PB). Next, specimens were dehydrated through an ascending ethanol series, starting with 50% ethanol, followed by incubation in 70% ethanol, 1% uranyl acetate (#21447, Polyscience Europe, Heidelberg, Germany), and 1% phosphotungstic acid (#455970, Merck, Darmstadt, Germany) solution overnight at 4 °C. Dehydration continued with an ascending ethanol series (80–100%). Afterward the samples were first incubated in propylene oxide (#807027, Merck, Germany), followed by an ascending series of propylene oxide and EPON mixtures. EPON consists of glycidether (#21045.02, Serva, Heidelberg, Germany), methylnadic anhydride (#29452.02, Serva, Heidelberg, Germany), 2-dodecenylsuccinic acid anhydride (#20755.01, Serva, Heidelberg, Germany) and 2,4,6-Tris(dimethylaminomethyl)phenol (#36975.01, Serva, Heidelberg, Germany) in a 5.4:3.8:1.84:1 mixture. The embedding procedure started with propylene oxide/EPON in a 3:1 ratio, followed by a 1:1 ratio, and ended with a 1:3 ratio. Finally, specimens were penetrated by pure EPON overnight at 20 °C. On the next day, EPON was renewed. The EPON-embedded specimens were allowed to polymerize at 60 °C for 2 days. 50 nm sections were cut with an Ultracut E Reichert-Jung (Leica Microsystems GmbH, Wetzlar, Germany) with a DiATOME histo diamond knife (45°, 6 mm, MX559; Diatome AG, Nidau, Switzerland), collected on Formvar-coated grids and contrasted with UranylLess (#22409, Electron Microscopy Sciences, Hatfield, USA) for 5 min. Samples were analyzed with a Zeiss LEO 910 transmission electron microscope equipped with a digital CCD camera. ImageJ 1.51 s (National Institutes of Health, Bethesda, MD, USA) was used for the evaluation of single mitochondrial morphological parameters (area, perimeter, circularity, Ferret’s diameter, aspect ratio, and roundness).

### Statistical analysis

Statistical analyses were performed using GraphPad Prism (vers. 9.3.1, San Diego, CA, USA). All data were tested for normal distribution using three normality tests (Anderson–Darling, D’Agostino–Pearson omnibus, and Shapiro–Wilk). Data were log-transformed when datasets were not normally distributed. In case, normal distribution was not achieved by log-transformation, non-parametrical tests were applied. Unpaired *t*-tests were applied to compare two groups, three or more groups were compared with a one-way ANOVA followed by Tukey’s or Dunnett’s multiple comparisons test, and two-factor analyses were conducted with a mixed-effects model followed by Šídák’s or Tukey’s multiple comparisons test. Sample sizes were chosen according to the recommendations of Naegle et al. [72] and are indicated in the respective figure legends. All data are expressed as mean ± SD. Statistical significance was defined as *p < 0.05; **p < 0.01, ***p < 0.001, and ****p < 0.0001.

### Supplementary information

Supplementary File 1

## Data Availability

Data generated and analyzed during the current study will be made available upon request.
