# Supplementary material for: Cigarette smoke causes a bioenergetic crisis in RPE cells involving the downregulation of HIF-1α under normoxia
Source: Cell Death Discov. 2023 Oct 25;9:398. doi: 10.1038/s41420-023-01695-5 (PMC10600121; doi:10.1038/s41420-023-01695-5)

Supplementary File 1

Cigarette smoke causes a bioenergetic crisis in RPE cells involving the downregulation of HIF-1α under normoxia

Figure 2A

HIF-1α

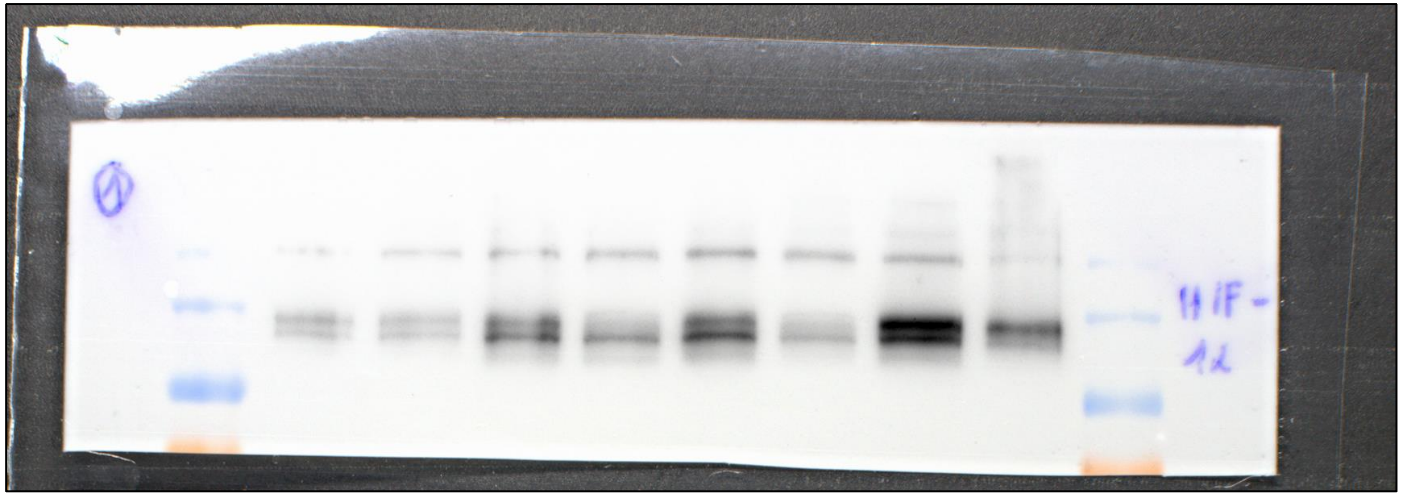

Tubulin

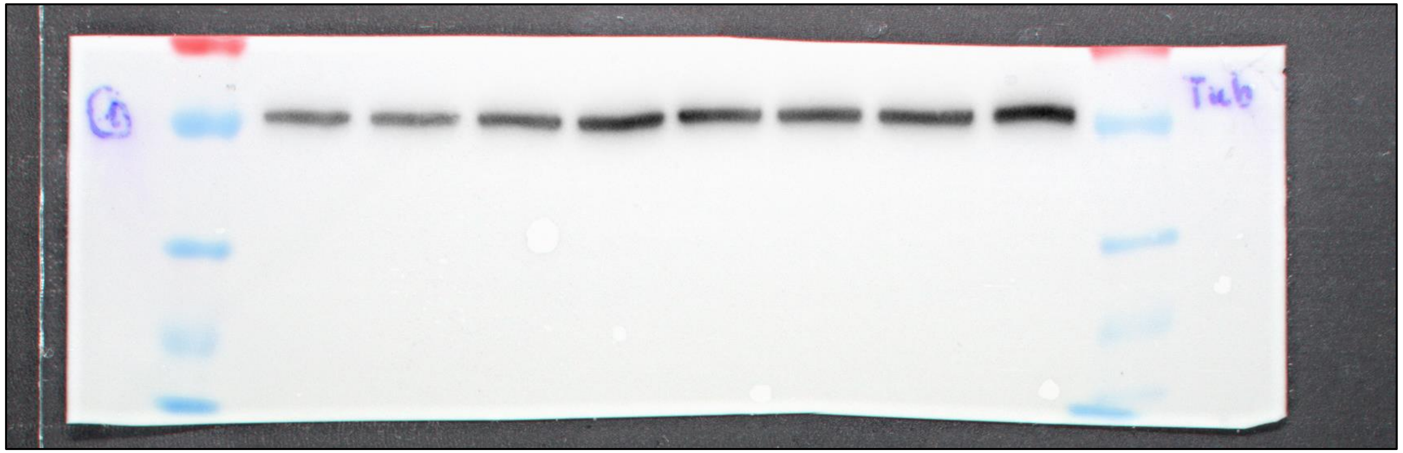

Figure 2B

Hydroxy-HIF-1α

Tubulin

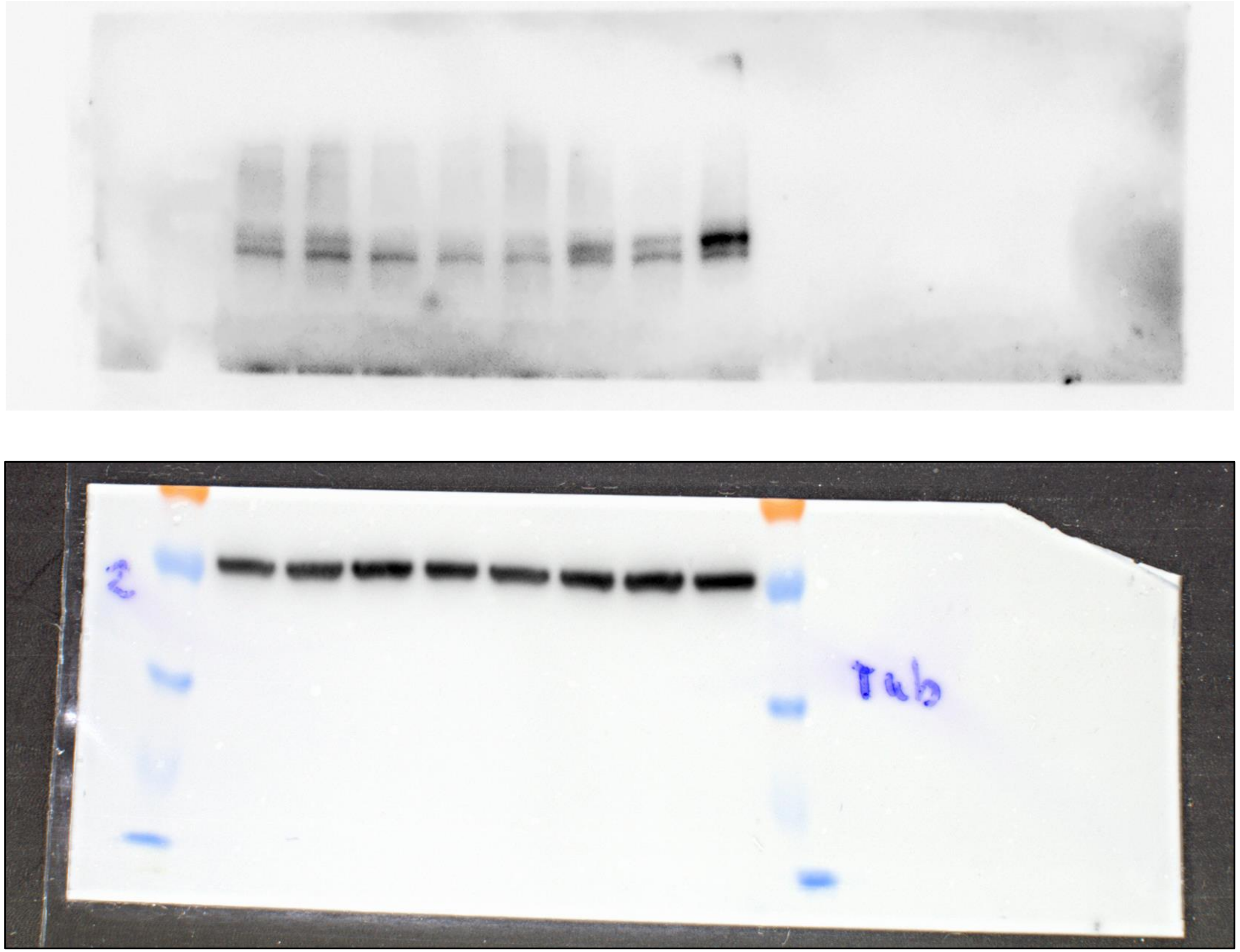

Figure 2C

HIF-2α

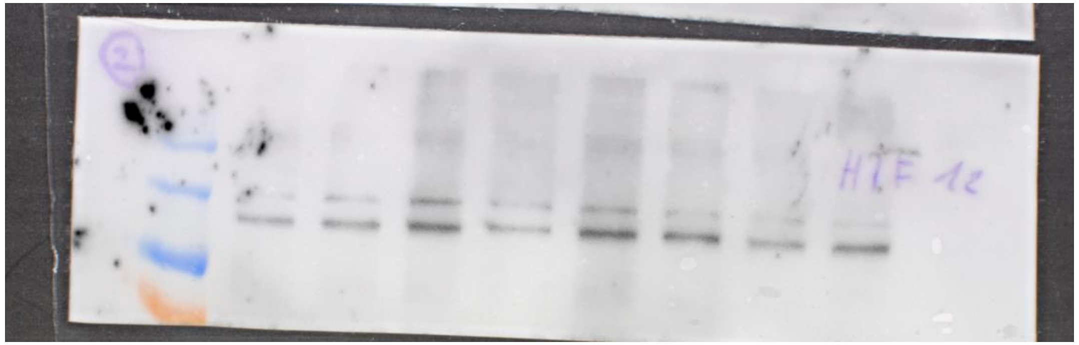

Tubulin

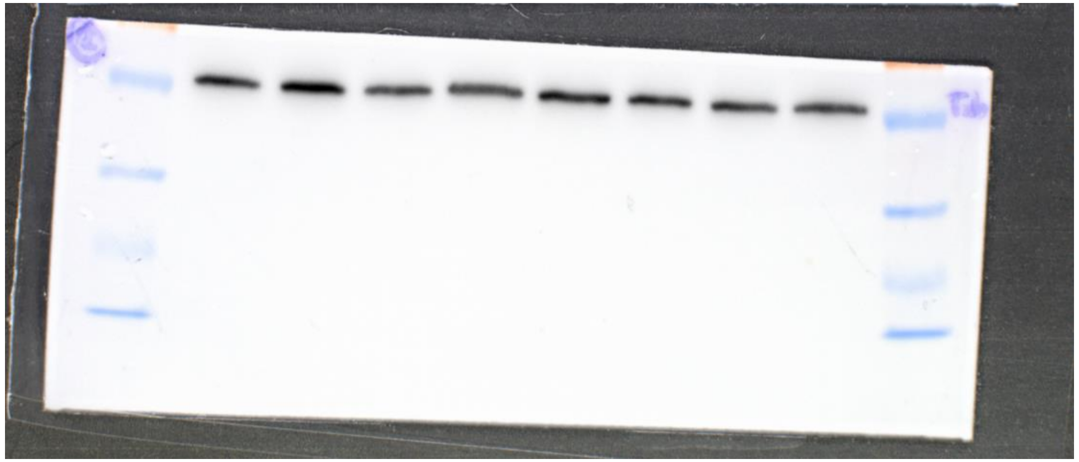

Figure 2D

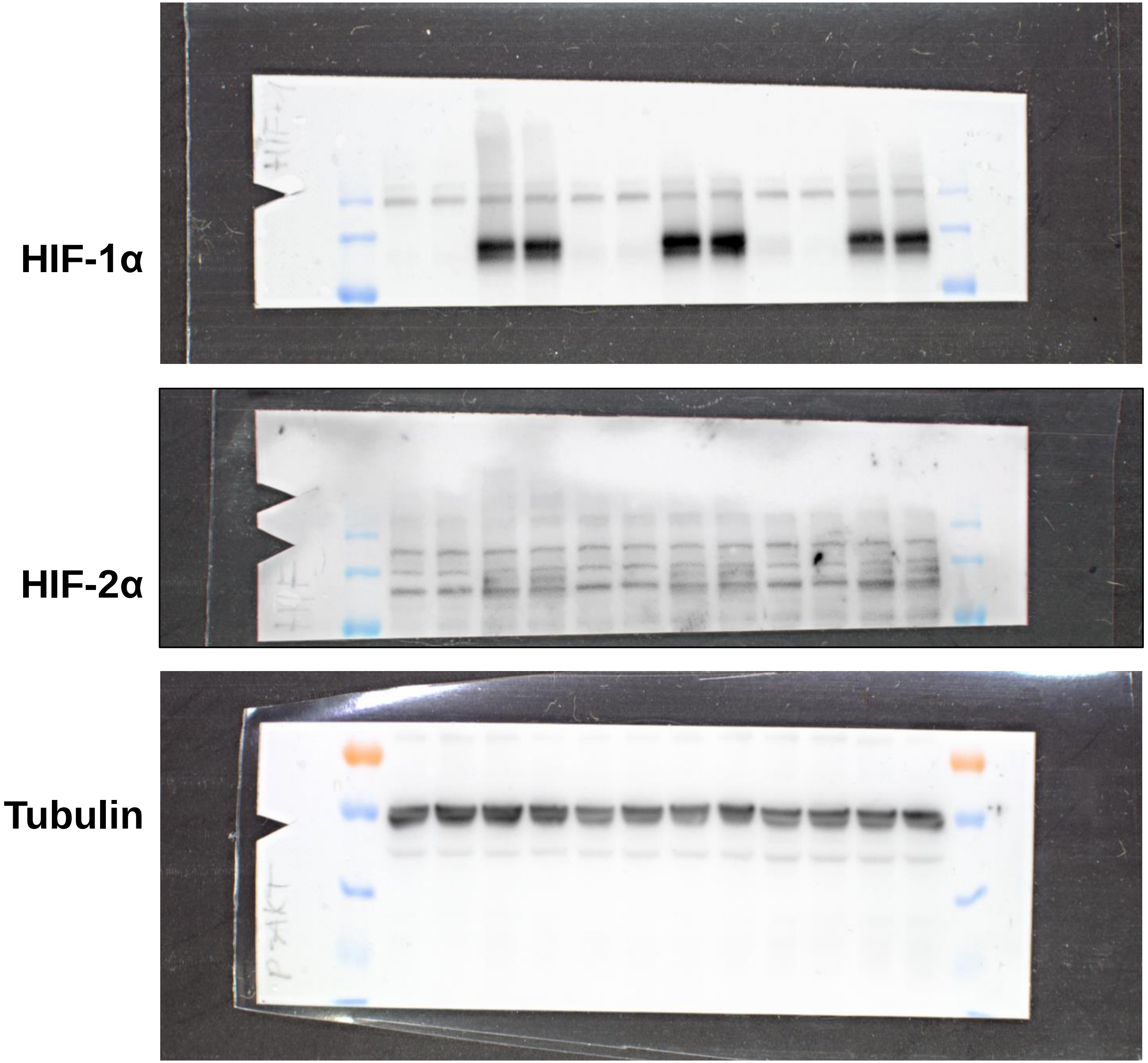

Figure 2E

HIF-1α

Hydroxy-HIF-1α

Tubulin

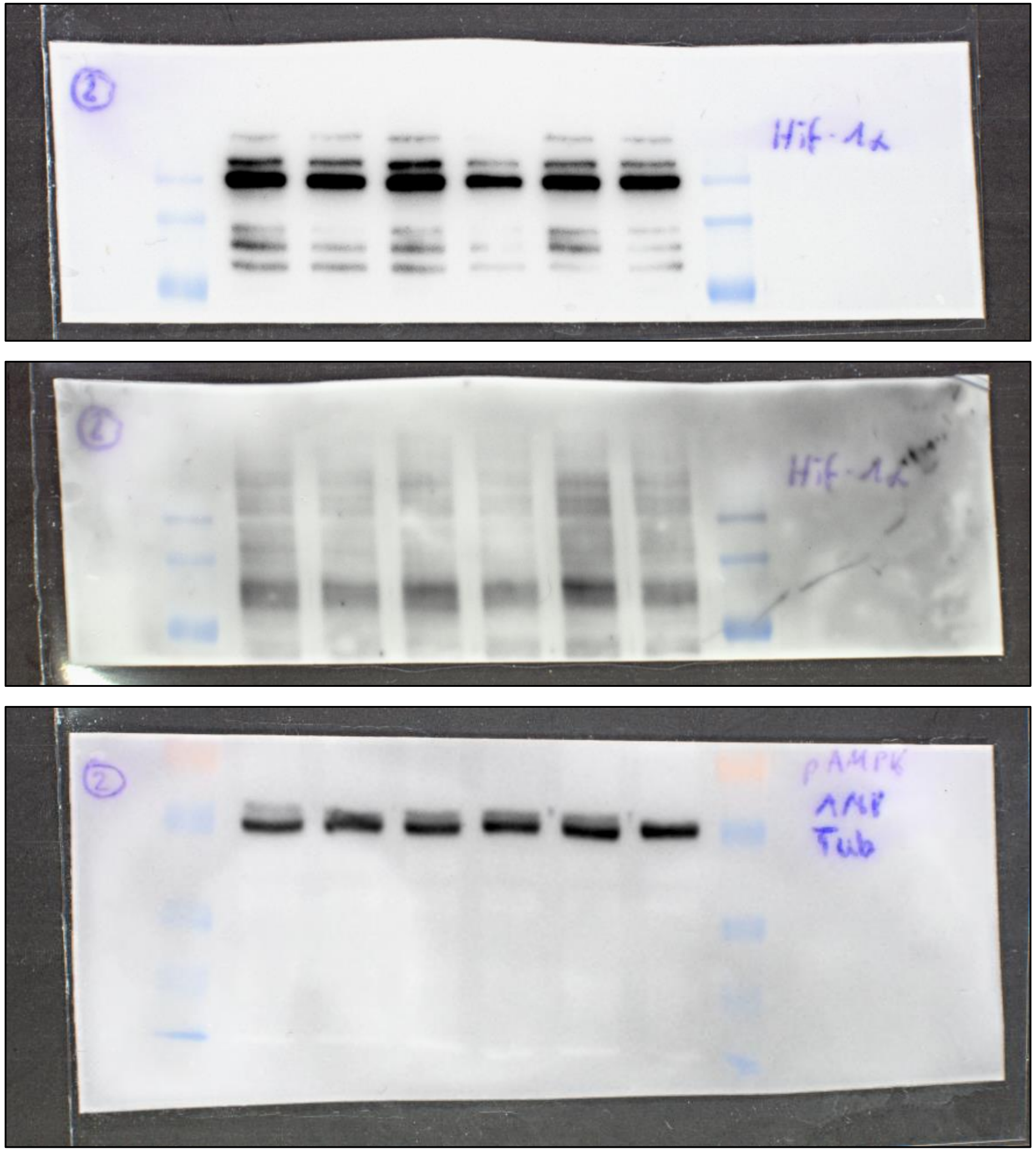

Figure 3K

TOM20

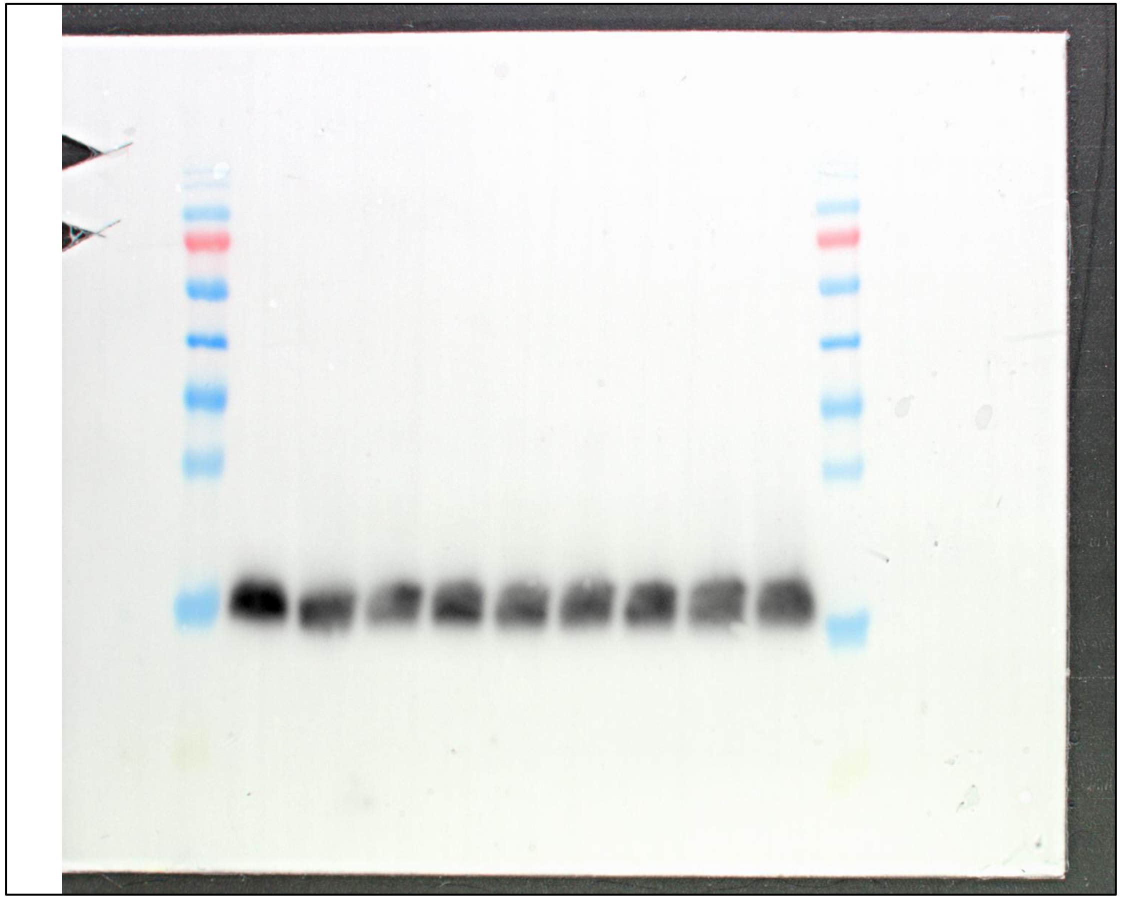

Tubulin

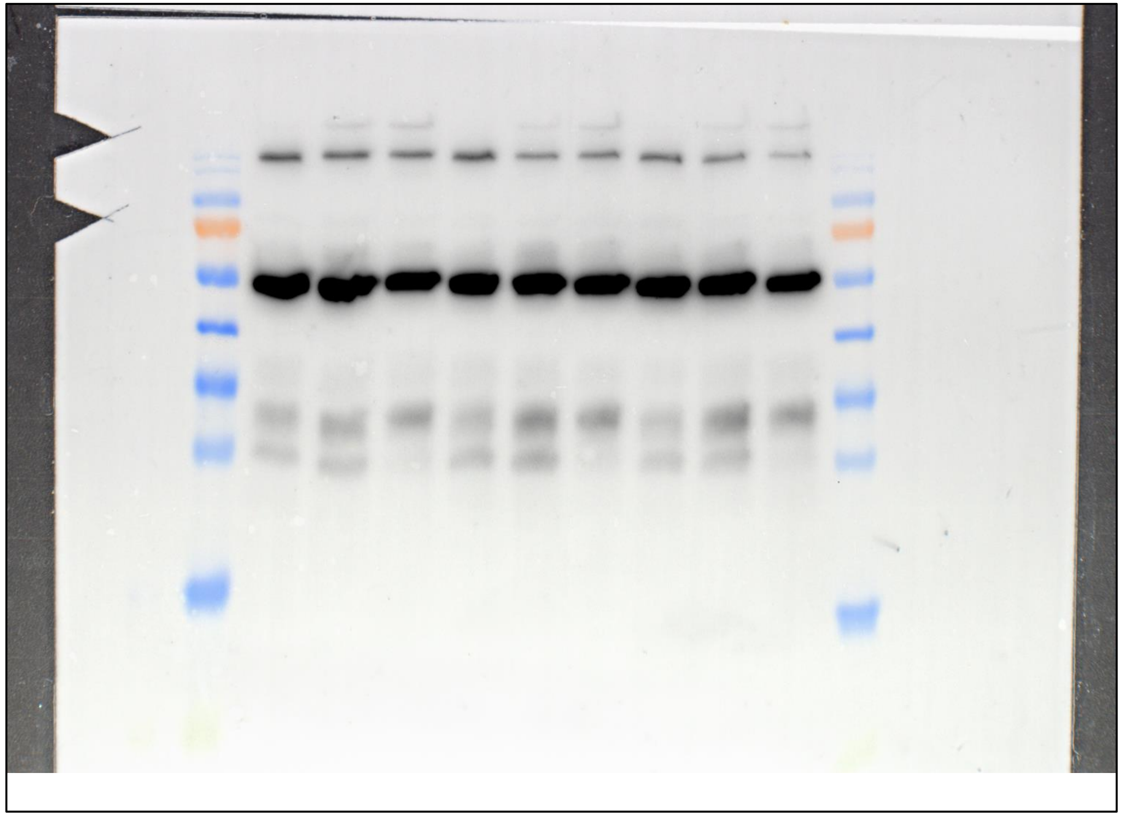

Figure 3K

HSP60

Unspecific band

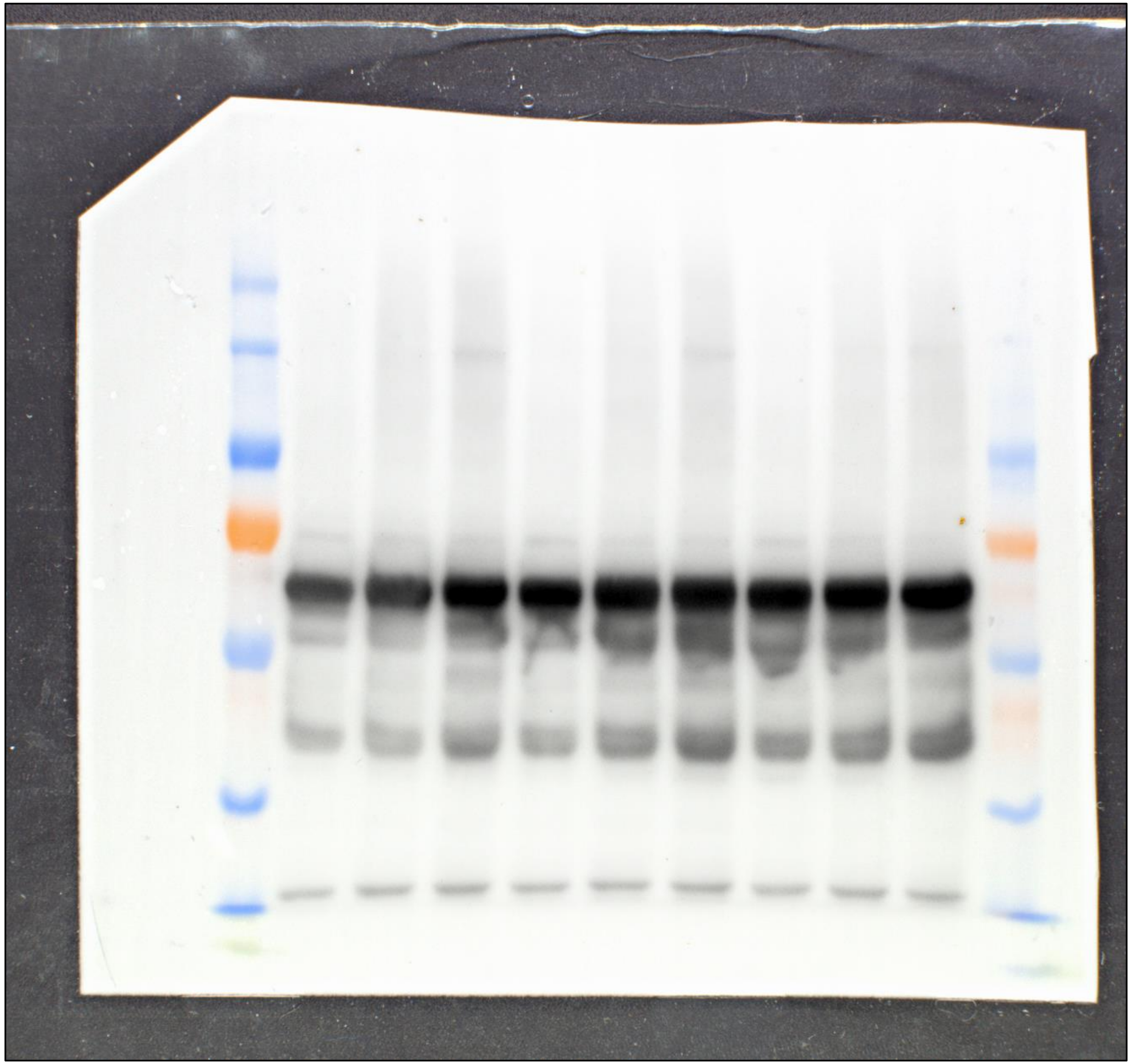

Figure 4E

HIF-1α

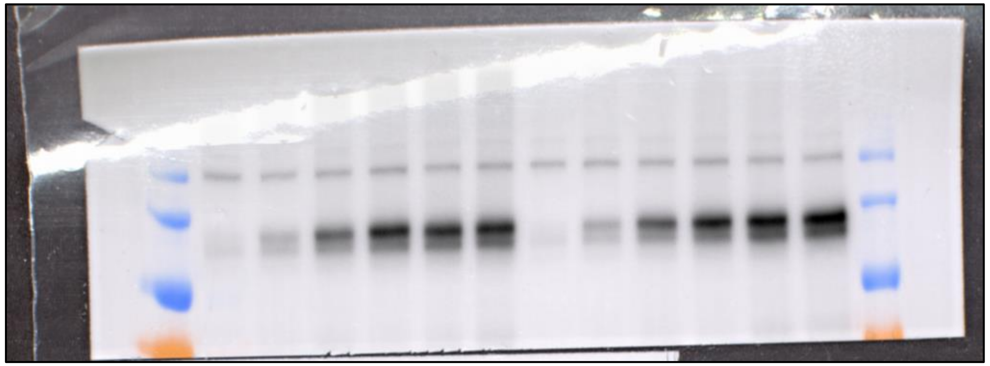

HIF-2α

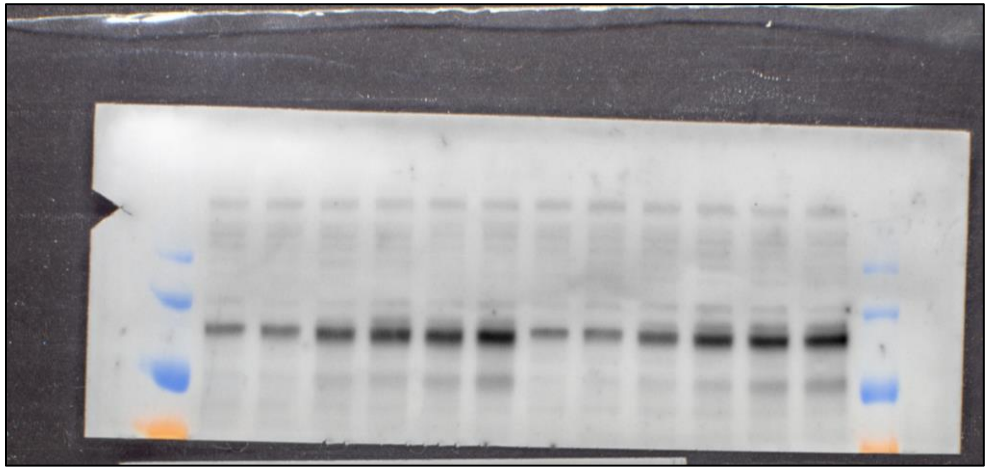

Tubulin

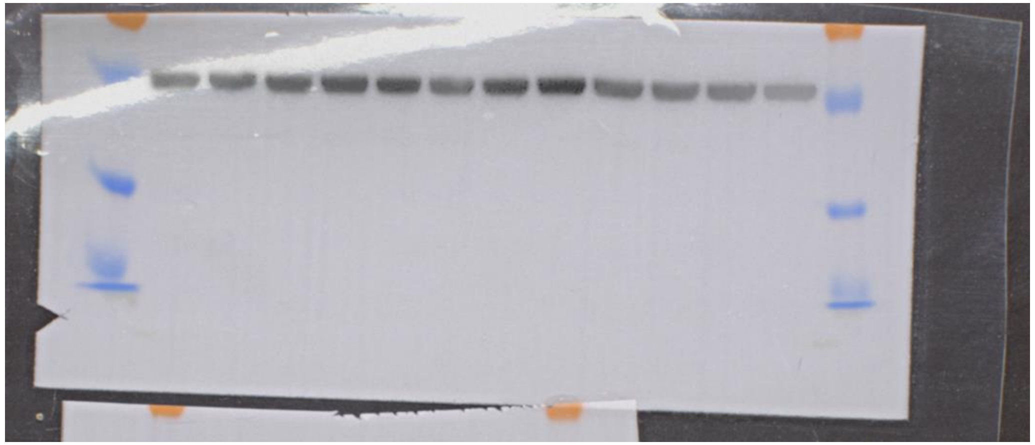

Supplement: Supplementary file 1 [file 41420_2023_1695_MOESM1_ESM.pdf]
